# A High-Performance MoS_2_-Based Visible–Near-Infrared Photodetector from Gateless Photogating Effect Induced by Nickel Nanoparticles

**DOI:** 10.34133/research.0195

**Published:** 2023-07-14

**Authors:** Ran Duan, Weihong Qi, Panke Li, Kewei Tang, Guoliang Ru, Weimin Liu

**Affiliations:** ^1^ State Key Laboratory of Solidification Processing, Center of Advanced Lubrication and Seal Materials, Northwestern Polytechnical University, Xi’an 710072, China.; ^2^ Shandong Laboratory of Yantai Advanced Materials and Green Manufacturing, Yantai 265503, China.; ^3^ State Key Laboratory of Solid Lubrication, Lanzhou Institute of Chemical Physics, Chinese Academy of Sciences, Lanzhou 730000, China.

## Abstract

Recent advancements in two-dimensional materials have shown huge potential for optoelectronic applications. It is challenging to achieve highly effective and sensitive broadband photodetection based on MoS_2_ devices. Defect engineering, such as introducing vacancies, can narrow the bandgap and boost the separation of photogenerated carriers by defect states but leads to a slow response speed. Herein, we propose a nickel nanoparticle-induced gateless photogating effect with a unique energy band structure to enable the application of defect engineering and achieve high optoelectronic performance. The device based on Ni nanoparticle-decorated MoS_2_ with S vacancies exhibited high responsivities of 106.21 and 1.38 A W^−1^ and detectivities of 1.9 × 10^12^ and 8.9 × 10^9^ Jones under 532 and 980 nm illumination (visible to near infrared), respectively, with highly accelerated response speed. This strategy provides new insight into optimizing defect engineering to design high-performance optoelectronic devices capable of broadband photodetection.

## Introduction

Recent advancements in two-dimensional (2D) materials have shown huge potential for optoelectronic applications [1–3]. Molybdenum disulfide (MoS_2_) is a representative 2D transition metal dichalcogenide that exhibits high carrier mobility [4], strong light absorption [5], and a layer-dependent bandgap of 1.2 to 1.8 eV (bulk to monolayer), of which the corresponding absorption spectrum spans from visible light to near infrared (NIR) [6,7]. Owing to these appealing properties, MoS_2_ has become a promising candidate for next-generation optoelectronic devices. Many impressive reports have shown the superior performance of MoS_2_-based photodetectors in the visible light regime. Yin et al. [8] first reported a monolayer MoS_2_ phototransistor that showed a responsivity of ≈7.5 mA W^−1^ along with a response time of less than 50 ms. Lopez-Sanchez et al. [9] demonstrated a phototransistor based on exfoliated monolayer MoS_2_ with an ultrahigh photoresponsivity of 880 A W^−1^ and a cutoff wavelength (λ) of ≈680 nm. However, the relatively large bandgap of MoS_2_ limits its optoelectronic applications in the NIR regime [10]. Some strategies shall be applied to enhance the performance of MoS_2_-based devices in the extended spectral wavelength regime.

Defect engineering, such as introducing S vacancies, provides promising insight into designing MoS_2_-based devices with broadband photodetection [11,12]. S vacancies can be easily introduced into MoS_2_ during synthesis or subsequent treatment with defect states localized between the valence band (VB) and conduction band (CB) and thus effective excitation under illumination of extended wavelengths [13,14]. For example, Xia et al. [10] demonstrated an enhancement of the photocurrent under NIR illumination after introducing S vacancies. Park et al. [15] fabricated a photodetector made from chemically exfoliated multilayer MoS_2_ with a photoresponsivity of 0.478 mA W^−1^ under 980 nm illumination because S vacancies were very likely formed during mechanical or chemical exfoliation [13]. Nevertheless, some unfavorable impacts of defect engineering need to be eliminated. For example, photogenerated carriers can be easily trapped by defect states, leading to a promotion of photogenerated carrier separation but a slow response speed and poor sensitivity [16].

Transition metal nanostructures, such as Au, Ag, and Pt, are usually placed on MoS_2_ to enhance light–matter interactions by localized surface plasmon resonance (LSPR) [17,18]. In fact, Ni, a low-cost 3d transition metal, can also strongly interact with MoS_2_ through interactions other than LSPR. By combining multilayer MoS_2_ crystals with Ni nanoparticles (Ni NPs), the hybrid system exhibits an optimized electrical performance [19]. Therefore, the photodetection applications of Ni NPs and MoS_2_ hybrid systems need to be unraveled. Herein, we present a Ni NP-decorated multilayer MoS_2_ crystal (Ni/MoS_2_) with S vacancies fabricated by wet impregnation and annealing in a reducing atmosphere and then its application as a high-performance photodetector with superior responsivity, sensitivity and response speed covering visible light and NIR. The S vacancies created by mechanical exfoliation were insufficient for effective NIR photodetection, and thus, annealing was used to create more S vacancies. Ni NPs induced a depletion of electrons and a gateless photogating effect, leading to high conductance for hole transport and hence a comprehensive improvement in optoelectronic performance. Our device showed a high responsivity and detectivity of 106.21 A W^−1^ and 1.9 × 10^12^, respectively, under 532 nm illumination. More importantly, our device showed superior NIR photodetection performance with a responsivity of 1.38 A W^−1^, a detectivity of 8.9 × 10^10^, and an external quantum efficiency of 165% under 980 nm illumination, outperforming a device based on MoS_2_ with S vacancies by approximately 10 times. This study proposed a new mechanism for metal nanoparticle–semiconductor hybrid systems. This strategy provides new insight into optimizing defect engineering and thus high-performance photodetectors capable of broadband photodetection.

## Results and Discussion

**Fig. 1.**
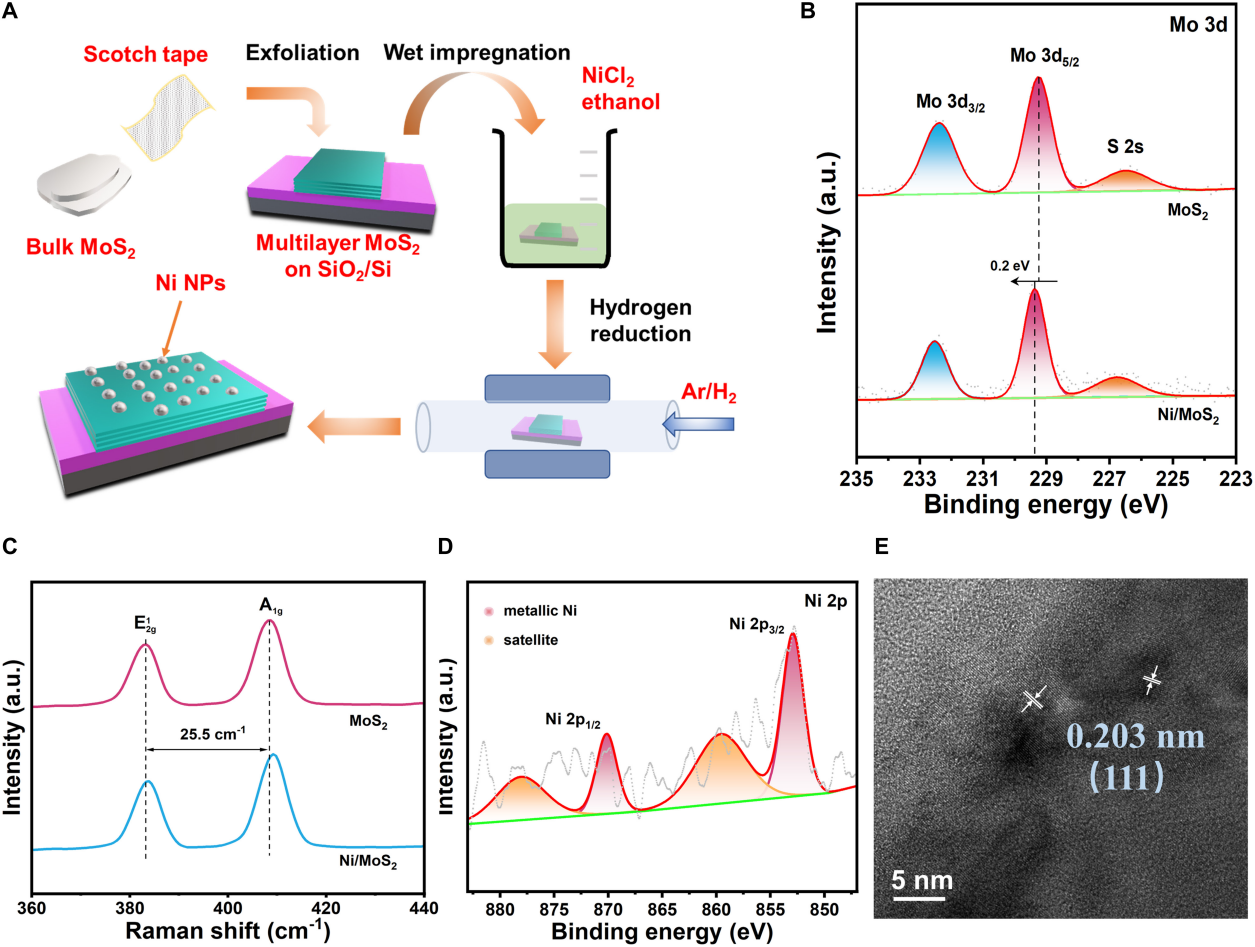
(A) Schematic illustration of synthesis of Ni/MoS_2_. (B) XPS spectra of Mo 3d and (C) Raman spectra of MoS_2_ and Ni/MoS_2_. (D) XPS spectrum of Ni 2p of Ni/MoS_2_. (E) HRTEM image of exfoliated Ni NPs.

The fabrication process of Ni/MoS_2_ is schematically illustrated in Fig. 1A. The details were explained in Materials and Methods. In brief, a multilayer MoS_2_ crystal was obtained by mechanical exfoliation using 3M scotch tape. In addition, the Ni NPs were placed on MoS_2_ by wet impregnation and hydrogen reduction in a tube oven with a flow of Ar/H_2_ (95%/5%). In addition, a multilayer MoS_2_ crystal annealed in reducing atmosphere without Ni precursor wet impregnation (named MoS_2_) was used as a contrast. X-ray photoelectron spectroscopy (XPS) was applied to further study the elemental compositions and electronic structures of MoS_2_ and Ni/MoS_2_. As shown in Fig. 1B, the peaks at 228.6 and 231.7 eV were related to the Mo 3d_5/2_ and Mo 3d_3/2_ of Mo^δ+^ (δ < 4), respectively, which demonstrated the formation of coordination unsaturated Mo sites due to the S vacancies [20,21]. After Ni NPs decoration, the peaks of Mo 3d negatively shift for ~0.1 eV. It indicated the charge transfer between MoS_2_ and Ni NPs due to the metal–semiconductor interactions [22]. More specifically, the electron migrated from Ni NPs to MoS_2_ owing to the higher electronegativity of Mo (2.16) than Ni (1.91) [23]. Figure 1C presents the Raman spectra of MoS_2_ and Ni/MoS_2_. The peaks at ≈383 and ≈409 cm^−1^ corresponded to the E^1^_2g_ and A_1g_ mode of MoS_2_, and the frequency difference = 25.5 cm^−1^ matched well with that of bulk MoS_2_ [24]. Ni 2p spectrum was presented in Fig. 1D to show the valence state of Ni NP. The peaks at 852.9 and 870.1 eV could be assigned to the metallic Ni with satellite peaks at 859.6 and 878.1 eV [25]. No signals of NiCl_2_ were detected due to the fully reduced Ni precursors by H_2_. Figure 1E presents the high-resolution transmission electron microscopy (HRTEM) image of Ni NPs exfoliated by sonication. The clear fringes with a distance of 0.203 nm were attributed to the (111) of fcc Ni, which further confirmed the formation of metallic nickel. We applied electron paramagnetic resonances (EPR) spectroscopy to verify the presence and concentration of S vacancies, because the peak intensity is strongly related to the dangling bonds originated from S vacancies [26]. The signals of Mo–S dangling bonds in Fig. S1 were clearly detected at *g* = 2.008 [27]. The mechanically exfoliated MoS_2_ without wet impregnation and annealing (pristine MoS_2_) showed a relatively lower concentration of S vacancies. The concentration of S vacancies barely changed after the formation of the Ni/MoS_2_ hybrid, which suggested that annealing could introduce extra S vacancies while the Ni NPs decoration induced no more S vacancies.

**Fig. 2.**
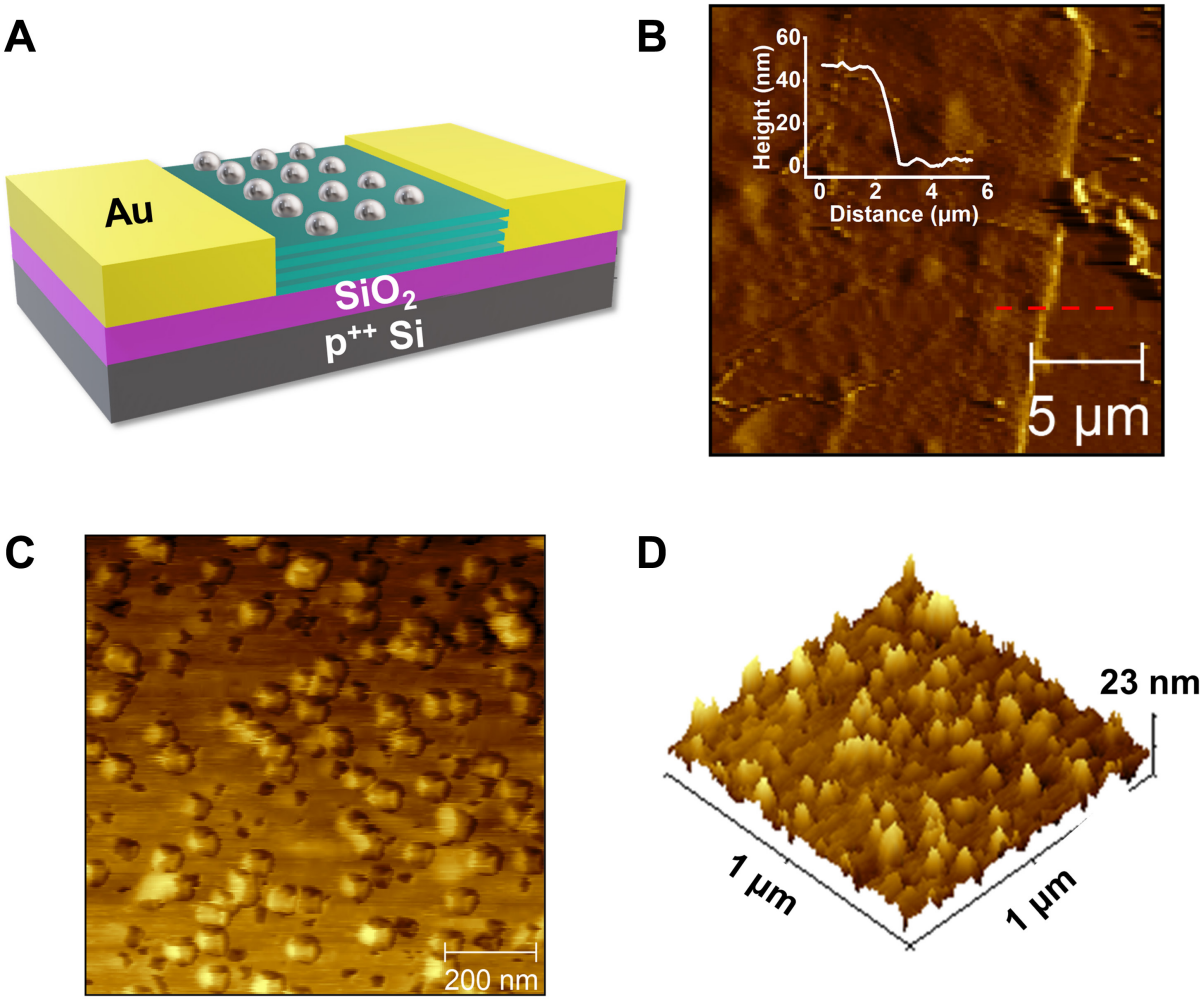
(A) Schematic illustration of the Ni/MoS_2_ device. (B and C) AFM images and corresponding (D) 3D view of Ni/MoS_2_.

Figure 2A illustrates the schematic model of the as-fabricated Ni/MoS_2_ photodetector. The thickness of MoS_2_ was ≈42 nm based on atomic force microscope (AFM) measurements, as shown in Fig. 2B. AFM topography image and 3D view displayed the surface morphology of Ni/MoS_2_. Figure 2C exhibits the surface of Ni/MoS_2_ covered by Ni NPs compared to the clean surface of MoS_2_ (Fig. S2), which confirmed the formation of Ni NPs and MoS_2_ hybrid. Note that the AFM image and signals in the 3D view (Fig. 2D) indicated that Ni NPs are polyhedral nanoparticles. The equilibrium shape of nanoparticle was often determined by the minimization of its surface free energy, which can be obviously reduced by contacting the substrate [28]. As a result of wet impregnation and growth on MoS_2_, Ni NPs could grow into nonspherical shapes. Figure S3A displays the scanning electron microscope (SEM) image of Ni/MoS_2_, from which mechanically exfoliated MoS_2_ uniformly covered with polyhedral nanoparticles can be clearly observed. The energy dispersive spectroscopy (EDS) was applied to measure the composition ratio of Ni/MoS_2_. The elemental mapping images in Fig. S3B–E exhibited the uniform distribution of Mo, S, and Ni. In addition, the S/Mo ratios of pristine MoS_2_, annealed MoS_2_, and Ni/MoS_2_ are shown in Fig. S4, respectively. The S/Mo ratio of pristine MoS_2_ (1.82) was larger than that (1.64) of annealed MoS_2_, which indicated that annealing introduced extra S vacancies into MoS_2_.

**Fig. 3.**
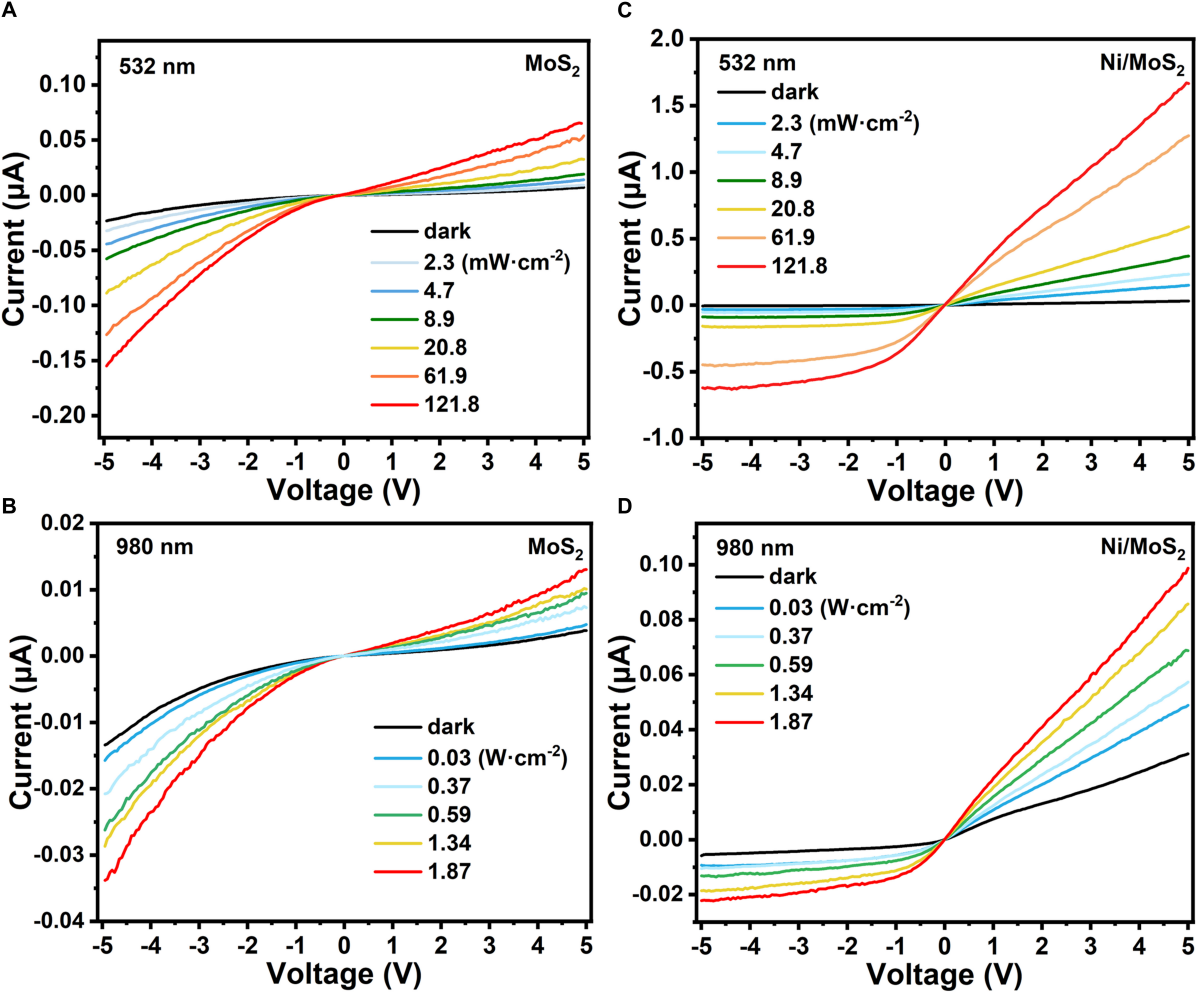
The *I*–*V* curves of the MoS_2_ device (A) under 532 nm illumination and (B) 980 nm illumination; the *I*–*V* curves of the Ni/MoS_2_ device (C) under 532 nm illumination and (D) 980 nm illumination.

The as-fabricated Ni/MoS_2_ device was utilized for broadband photodetector covering visible light and NIR. Fiber laser devices with a wavelength of 532 nm and 980 nm were used to conduct experiments. The current–voltage (*I*–*V*) characteristics of the as-fabricated photodetector (Ni/MoS_2_) and contrast photodetector (MoS_2_) under dark and illuminated conditions are presented in Fig. 3. Clearly, the current under illumination (*I*_light_) for the Ni/MoS_2_ device increased by 2 orders of magnitude than that of the MoS_2_ device, along with slightly increasing dark current (*I*_dark_) under positive voltage. In the *I*–*V* curves, the device showed a depletion of electrons (*I*_*V=*5V_*/I*_*V=−*5V_ ≈ 5.7, which is the ratio of current at *V* = ±5 V under dark conditions), which indicated a depletion of electrons and a photogating effect referring to other devices modulated by gate electrode or partially doping [29].

Density functional theory (DFT) calculations were performed to investigate the influence of the S vacancies on the energy band structure and spectral absorption of MoS_2_. The plots of total density of states of intact MoS_2_ and MoS_2_ with S vacancies were presented in Fig. S5A and B. When S vacancies were introduced into the MoS_2_ supercell, the energy band structure changed. Defect states emerged in the forbidden gap region near the conduction band leading to a narrower bandgap. In addition, the corresponding calculated absorption spectra are shown in Fig. S5C and D. No absorption of NIR was found in the absorption spectrum of intact MoS_2_ due to the wide bandgap of MoS_2_ and thus the short cutoff wavelength. After the introduction of S vacancies, an obvious absorption peak of approximately 980 nm was observed in the NIR region. The calculations could demonstrate that the S vacancies in MoS_2_ played a critical role in the effective NIR photodetection.

**Fig. 4.**
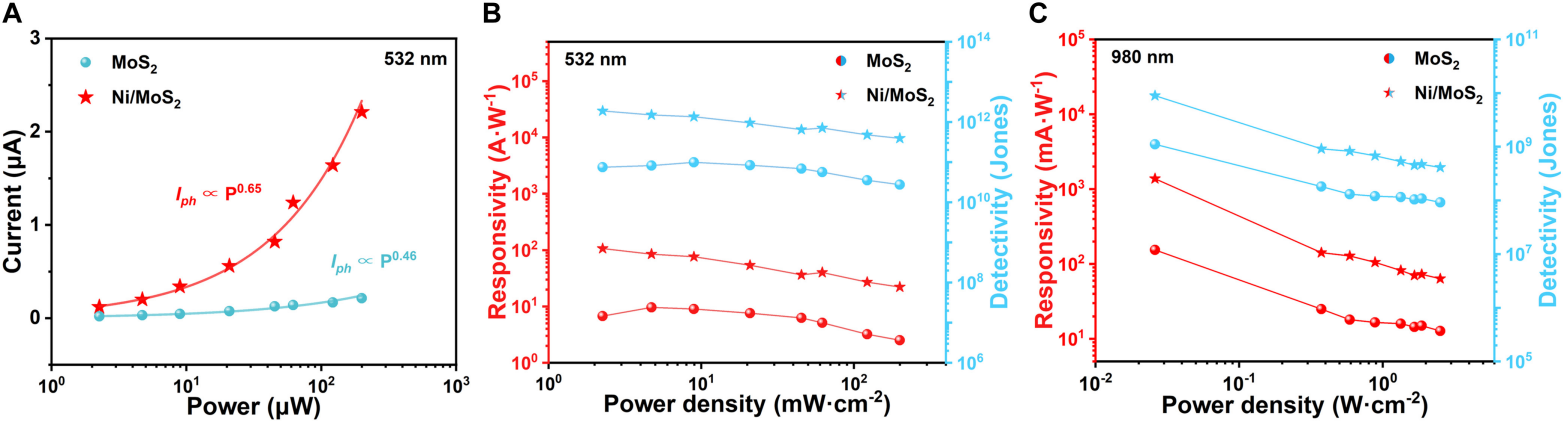
(A) Photocurrent as a function of the incident light density under 532 nm illumination at ±5 V. (B) Responsivity and detectivity of the MoS_2_ device and the Ni/MoS_2_ device under 532 nm illumination at ±5 V. (C) Responsivity and detectivity of the MoS_2_ device and the Ni/MoS_2_ device under 980 nm illumination at ±5 V.

Figure 4A shows the photocurrent *I*_ph_ (|*I*_light_ *− I*_dark_|) as a function of various values of incident light power (*P*). The *I*_ph_ increases with *P* due to increasing photogenerated carriers, satisfying *I_ph_* ∝ *P^α^* (α denotes an empirical value), and the value of α is expected to be 1 in the ideal case [30]. The α is related to the long-lived trap states induced by photogenerated carrier recombination [31]. The α of MoS_2_ and Ni/MoS_2_ devices was 0.46 (at *V* = −5 V) and 0.65 (at *V* = +5 V), respectively. The increasing α after Ni NPs decoration demonstrated the suppression of trap-assisted recombination, which was imperative for the performances of defect-engineered optoelectronic devices. Then, we evaluated the optoelectronic performances of MoS_2_ and Ni/MoS_2_ by calculating the responsivity (*R*), detectivity (D^*^), and external quantum efficiency (*EQE*). The key parameters for devices are as follows:

$$R(A/W)=\frac{I_{\mathrm{ph}}}{P}$$ (1)

$$D^{*}(\text{Jones})=\frac{RA^{1/2}}{{\left(2eI_{\text{dark}}\right)}^{1/2}}$$ (2)

and

$$\mathrm{EQE}(\%)=\frac{\mathrm{Rhc}}{e\lambda }\times 100$$ (3)

where *A* is the effective area of device, *e* is the unit charge, *h* is Planck’s constant, *c* is the speed of light, and *λ* is the wavelength of incident light. As shown in Fig. 4B, under 532 nm illumination with a power density of 2.3 μW cm^−2^, the Ni NPs decoration led to *R* increasing from 6.75 A W^−1^ (MoS_2_ device) to 106.21 A W^−1^ (Ni/MoS_2_ device), D^*^ increasing from 7.4 × 10^10^ to 1.9 × 10^12^, and *EQE* increasing from 2.2 × 10^3^% to 2.5 × 10^4^%, respectively. More impressively, under 980 nm illumination with a power density of 25.9 μW cm^−2^, the Ni/MoS_2_ device showed an improvement of *R* from 0.15 A W^−1^ (MoS_2_ device) to 1.38 A W^−1^ (Ni/MoS_2_ device), D^*^ from 1.1 × 10^9^ to 8.9 × 10^9^, and *EQE* from 36.8% to 167% (Fig. 4C). We also presented the performance under 980 nm illumination of mechanically exfoliated MoS_2_ without wet impregnation and annealing (pristine MoS_2_) in Fig. S6 to demonstrate the optimization on *R* and D^*^ resulting from introducing more S vacancies by annealing. The mechanical exfoliation could create S vacancies, but it is uncontrollable and insufficient for effective photodetection under NIR illumination. The comprehensive performances of Ni/MoS_2_ are better than most reported MoS_2_-based devices, as shown in Table. The Ni/MoS_2_ device showed a comprehensive improvement in each key parameter due to the depletion of electrons and photogating effect, which led to a high conductance for hole transport.

**Table.** Comparison of characteristic parameters for MoS_2_-based photodetectors.

Materials	Wavelength (nm)	Responsivity (mA W^−1^)	EQE (%)	Detectivity (Jones)	Ref.
Ni/MoS_2_	980	1380	165%	8.9 × 10^9^	This work
Au/MoS_2_	980	64	NA	NA	[43]
Au/MoS_2_	780	61.13	9.71	3.53 × 10^10^	[44]
Pt/MoS_2_	780	42.3	6.72	1.83 × 10^10^	
Pd/MoS_2_	780	32.7	5.53	1.63 × 10^10^	
Au/MoS_2_	532	24	NA	5.3 × 10^9^	[17]
Ag/MoS_2_	980	0.881	NA	1.28 × 10^9^	[45]
NaYF4:Yb/Er@MoS_2_	980	0.166	0.021	5.6 × 10^6^	[46]
MoS_2_–CQD	780	2.62	NA	NA	[47]
MoS_2_/Si	850	10.07	NA	4.53 × 10^10^	[48]
Graphene/MoS_2_/graphene	904	23.5	NA	NA	[49]
WS_2_/MoS_2_	850	710	NA	4.4 × 10^9^	[50]

**Fig. 5.**
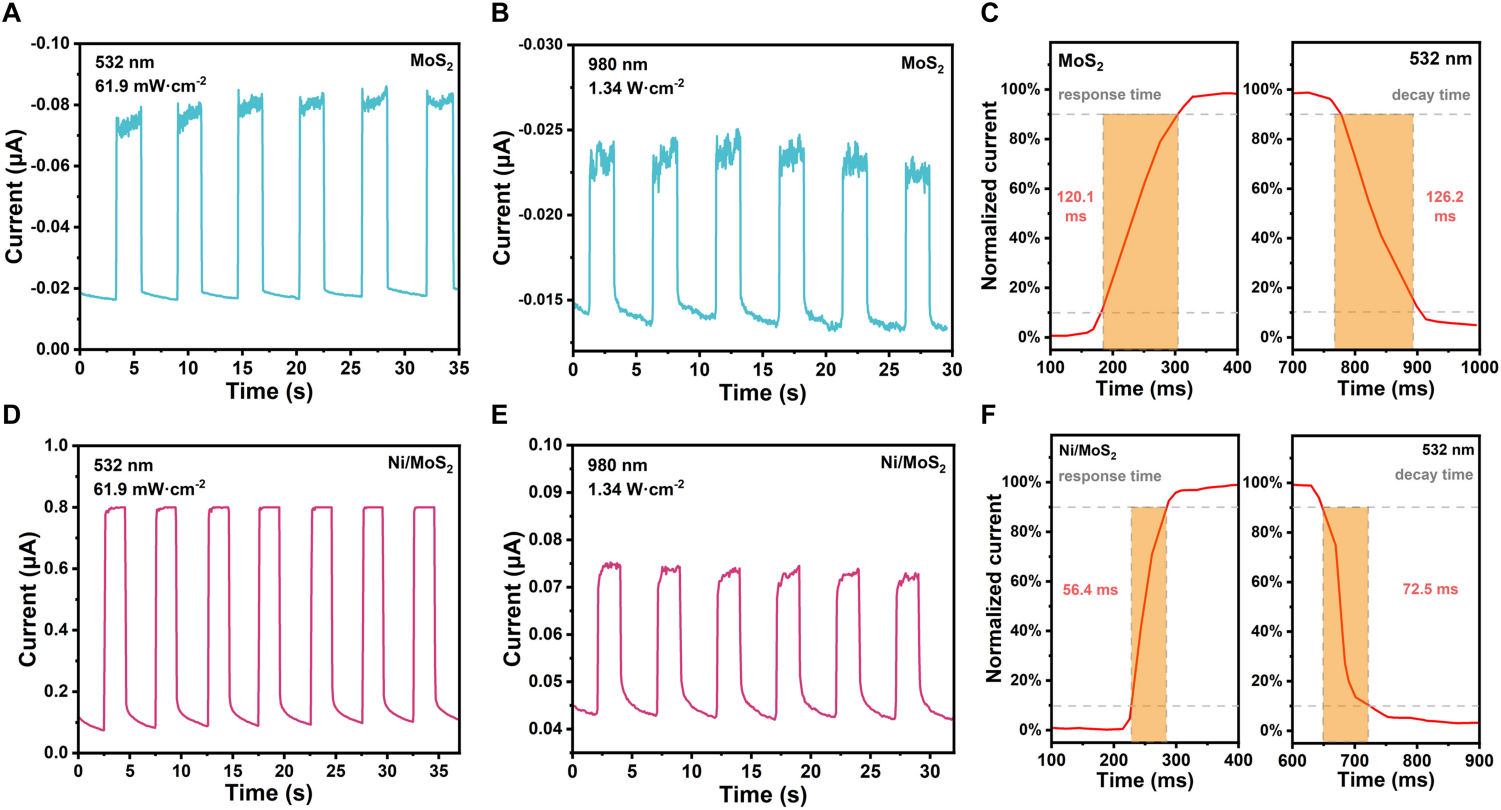
The time-dependent photoresponse of the MoS_2_ device under (A) 532 nm illumination with a power density of 61.9 mW cm^−2^ and (B) 980 nm with a power density of 1.34 W cm^−2^. (C) Magnified plots of the time-dependent photoresponse of the MoS_2_ device under 532 nm illumination. The time-dependent photoresponse of the Ni/MoS_2_ device under (D) 532 nm illumination with a power density of 61.9 mW cm^−2^ and (E) 980 nm with a power density of 1.34 W cm^−2^. (F) Magnified plots of the time-dependent photoresponse of the Ni/MoS_2_ device under 532 nm illumination.

Figure 5 shows time-resolved photoresponse under 532 nm and 980 nm illumination. The Ni/MoS_2_ device exhibited a highly stable and reversible response compared to the rather unstable response of the MoS_2_ device. The magnified photoresponse cycles in Fig. 5C and F of the Ni/MoS_2_ device exhibited a response/decay time (*τ*_rise_/*τ*_decay_) of 50.3/56.6 ms under 532 nm illuniation, which was shorter than that of MoS_2_ (120.1/126.2 ms) under 532 nm illuniation. The *τ*_rise_*/τ*_decay_ was strongly related to defect states. The trapped electrons reduced the conductance in the channel, and S vacancy as a active site for persistent oxygen abosorption and disorption caused a quite long response time [32,33]. After turning off the light, many holes had already recombined with the electrons at defect states while there were more electrons remaining in the channel leading to a long decay time to reach equilibrium state [34,35]. The Ni NPs could enhance the channel conductance and holes could recombine with electrons at the interface between Ni and MoS_2_ (mechanism was explained in the follwing). Therefore, the device more easily reached equilibrium state whether under dark or illumination conditions.

**Fig. 6.**
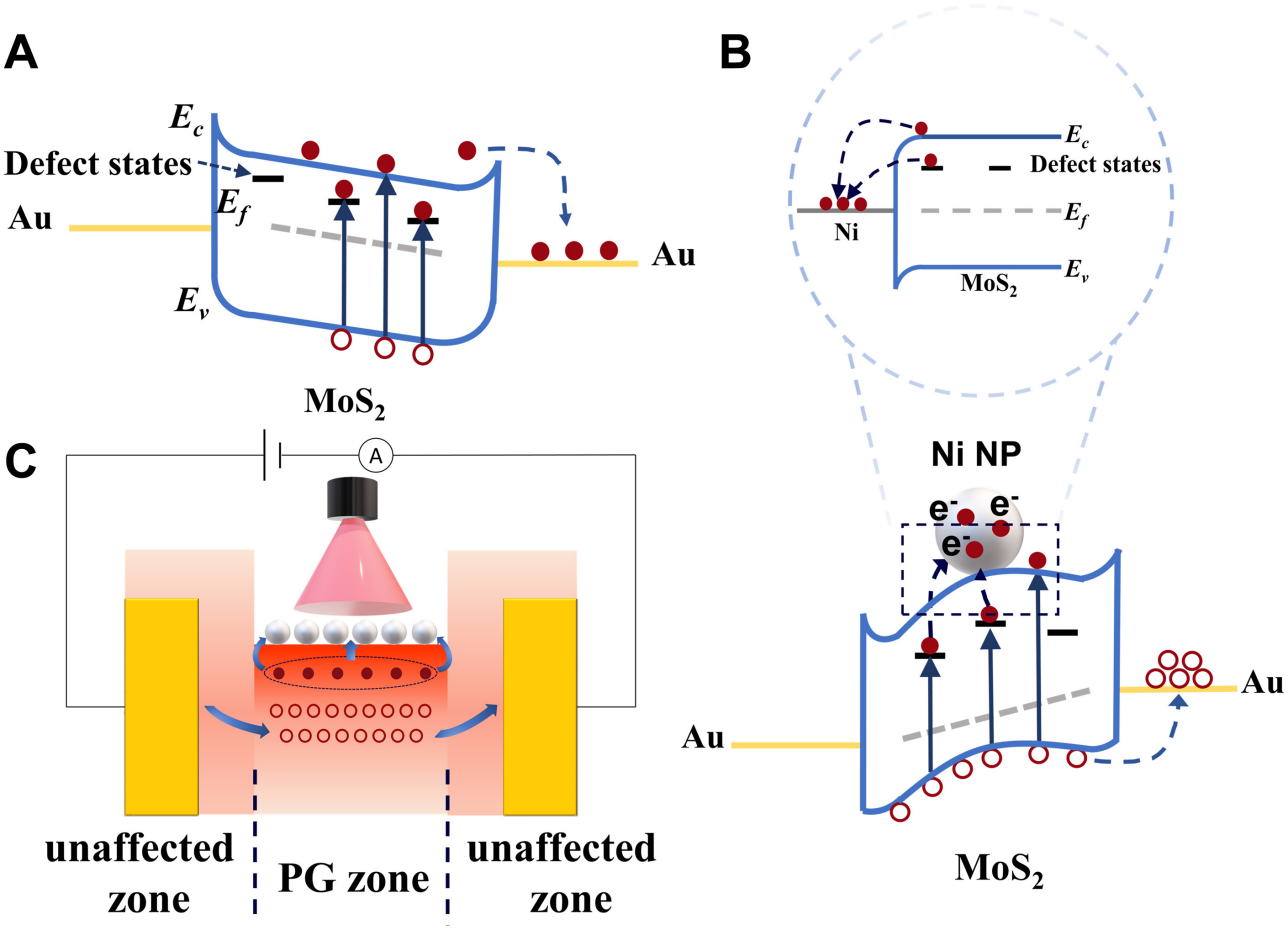
(A) Energy band diagram of the MoS_2_ device under negative voltage. (B) Energy band diagram of the Ni/MoS_2_ device under positive voltage and the zoom-in energy band structure of Ni NP and MoS_2_. (C) Schematic illustration of the transport behavior of the Ni/MoS_2_ device under illumination.

The energy band diagrams of devices are displayed in Fig. 6 to illustrate the mechanism of the optimization introduced by Ni NPs decoration. Work function of Au is very close to that of Ni (Φ_M-Au_ = 5.1 eV and Φ_M-Ni_ = 5.1 eV) [36], and the electron affinity and bandgap (*E_g_*) of MoS_2_ are ~4.2 and ~1.2 eV, respectively [37]. A typical energy band structure (Fig. 6A) was formed as previously reported [29]. After Ni NPs decoration, electrons in the channel near Ni NPs spontaneously transfered to Ni NPs powered by photovoltaic effect (Fig. 6B) [38]. Then, the Schottky barrier for electrons hindered the further flowing-out of electrons and hence electrons accumulating in Ni NPs. Therefore, Ni NPs acted as a gate electrode (gate voltage<0) optimizing the channel conductance, leading to the high conductance for hole transport and suppressing the trap-assisted recombination, eventually leading to improvement in all aspects. Although Ni NPs could act as a gate electrode, it was actually a localized effect unlike a real gate electrode providing stable voltage. The region of MoS_2_ contacting the Au electrode was independent of the Ni NPs-induced photogating effect because the circuit from MoS_2_ to Ni NPs was shorted while under this circumstance, Au/MoS_2_ and Ni/MoS_2_ were parallel, as illustrated in Fig. 6C. The absorption and desorption process of ambient molecules on the S vacancies could continuously generate current to delay the process by which the device reached equilibrium state, thus a prolonged response time [35]. Note that Ni NPs depleted the electrons in the channel and at the trap centers, which accelerated the process of reaching equilibrium state, and hence a reduced response time. Moreover, the process of electrons flowing into Ni NPs adjusted the number of remaining electrons and holes in MoS_2_. In the channel of MoS_2_, holes became the majority carrier since electrons in the channel were depleted due to the photovoltatic effect of Ni/MoS_2_. Therefore, the trap-assisted recombination was replaced by a new process in which the remaining holes in the channel could recombine with the electrons in the Ni NPs at the interface under dark conditions, which contributed to a reduced decay time [33]. The relationship of current and *P* of the Ni/MoS_2_ device at *V* = −5 V and the corresponding energy band diagrams are illustrated in Fig. S7. The value of *α* was 0.76, larger than that of the MoS_2_ device (0.46) at *V* = −5 V, which indicated a suppression of trap-assisted recombination under illumination due to many electrons flowing to Ni NPs. Due to the high Schottky barrier for electrons and the low conductance for electron transport induced by Ni NPs, few electrons flew in the channel and thus a low current under negative voltage (Fig. S7B) [39].

To demonstrate the mechanism of the Ni/MoS_2_ device, we had to confirm the unreplacable importance of Ni NPs. In fact, a thin oxidation layer of Ni NPs could prevent Ni NPs from the absorption of oxygen molecules. However, if some Ni NPs were severely oxidized by oxygen in the air and this eventually eroded the interface of Ni NPs and MoS_2_, a high Schottky height (MoS_2_/NiO heterojunction) for electrons flowing out from MoS_2_ to Ni was going to be built as illustrated by previous reports [40]. This could result in a weakened photovoltatic effect of Ni NPs/MoS_2_ due to the photogerated electrons partially combining with oxygen molecules and less electrons flowing into Ni NPs due to the high Schottky height of the MoS_2_/NiO heterojunction. The Ni/MoS_2_ device was exposed to air for 1 month for oxidation. The photoresponse measurements under 980 nm illumination (strongly influenced by defect states) were conducted and are presented in Fig. S8. After exposure to air, the photocurrent drastically decreased due to the absorption of oxygen molecules on the vacancies, while the ratio of *I*_*V=*5V_/*I*_*V=−*5V_ decreased from 5.3 (before exposure) to 3.2 (after exposure), suggesting the weakened effect of depletion of electrons and thus lower Schottky height for electrons. This result was exactly consistent with our assumption. The absorption of oxygen molecules also led to the low value of α ≈ 0.56 at *V =* 5 V owing to the weak photogating effect and persistent recombination at trap centers. Then, the Ni NPs were eleminated by hydrochloric acid. As shown in Fig. S9, the transport behavior significantly changed and the depletion of electrons was barely observerd. The values of current under positive and negative voltage were almost equal, because the depletion of electrons and the photogating effect by Ni NPs decoration were eleminated when Ni NPs were fully etched. The photogating effect was now purely induced by S vacancies, which was far more weaker than Ni NPs. These results could confirm that the Ni NPs are definitely the origin of the depletion of electrons and photogating effect.

## Conclusion

In conclusion, we successfully demonstrated a significant enhancement in optoelectronic performance by defect engineering (introducing S vacancies) and decoration of Ni nanoparticles on a MoS_2_ multilayer photodetector. S vacancies could lead to effective photodetection under NIR. Due to the photovoltaic effect, the electrons in the channel could flow into the Ni NPs, making the Ni NPs act as a gate with a negative voltage. The photogating effect of Ni NPs was found to lead to a suppression of trap-assisted recombination and a high conductance for hole transport, thus optimizing the sensitivity, responsivity, and response speed of the Ni/MoS_2_ device under NIR illumination. This study proposes a mechanism for metal–semiconductor hybrid photodetectors apart from LSPR. This strategy could minimize the unfavorable impacts of defect engineering enabling MoS_2_-based optoelectronic devices for high-performance broadband photodetection.

## Materials and Methods

### Materials synthesis

A commercial MoS_2_ crystal (SUNANO GROUP) and 3M Scotch tape were used for the repeated peeling of MoS_2_ into nanosheets. Before transfer, SiO_2_/Si was sonicated in acetone, ethanol, and deionized water (> 18 MΩ cm resistivity) for 10 min each. Then, MoS_2_ nanosheets were transferred onto polydimethylsiloxane and placed over the cleaned SiO_2_/Si. After 10 min of heating at 85 °C, MoS_2_ nanosheets could be deposited on SiO_2_/Si. The wet impregnation method was applied to cover MoS_2_ nanosheets with Ni^2+^. NiCl_2_·6H_2_O (23.7 mg) was mixed with 10 ml of ethanol and sonicated for 10 min to form a uniform solution (called solution A). The as-fabricated MoS_2_ nanosheets on SiO_2_/Si were immersed in solution A for 2 h to allow the placement of Ni^2+^ on MoS_2_ nanosheets. After wet impregnation, MoS_2_ sheets were annealed at 400 °C in an Ar/H_2_ flow (95%/5% in volume ratio) with a flow speed of 60 sccm for 2 h with a heating rate of 10 °C min^−1^. The MoS_2_ nanosheets used in the MoS_2_ device as a contrast were prepared via the same route except for wet impregnation.

### Materials characterization

AFM (MFP 3D Origin+) was employed to characterize the thickness of the nanosheets. HRTEM was conducted on an FEI Talos F200x. SEM and EDS were conducted on a TESCAN MIRA3 equipped with energy-dispersive x-rays. XPS spectra were measured on a Thermo ESCALAB 250XI system. Raman spectra were obtained using a confocal microscope-based Raman spectrometer (Alpha300R) with an excitation laser of 532 nm.

### Device fabrication and photoresponse measurements

Two devices were fabricated on SiO_2_/Si with 300 nm of SiO_2_ as the insulating layer. Au electrodes were defined via 3 steps: e-beam lithography, Au electrode deposition, and a lift-off process. The photoresist layer (ROL-7133) was coated by a spin-coater. Patterning was completed using e-beam lithography (Midas MDA-400M) with a 350-W ultraviolet (UV) source, and the substrates were exposed to UV light for 5 s and then developed in a ZX-238 solution for 20 s. The as-fabricated Ni/MoS_2_ and MoS_2_ nanosheets were transferred onto Au electrodes by polymethyl methacrylate (PMMA). Acetone was used to remove residual PMMA from the devices. In addition, the devices were annealed at 300 °C under an Ar atmosphere for 2 h to relieve residual stress and eliminate the air gap between the materials and metal electrodes. To apply photoresponse measurements, the device was illuminated with laser power supplies of 532 nm and 980 nm (LSR-PS-II, LASERVER), and the data were recorded by using a source meter (Model 2450, Keithley).

### Computational methods

We calculated the density of states and absorption spectra of monolayer MoS_2_ and monolayer MoS_2_ with S vacancies by DFT using the Vienna Ab initio Simulation Package with the Perdew–Burke–Ernzerhof exchange-correlation function and the projector augmented wave method [41]. The energy cutoff was set to 500 eV. The electronic iteration convergence is 10^−5^ eV and 0.01 eV Å^−1^, using the normal (blocked Davidson) algorithm to optimize the geometry. The break criterion for the electronic self-consistent loop was set to 10^−5^ eV. The requested k-spacing is 0.25 Å^−1^, which leads to a 4 × 4 × 2 mesh for the density of states (DOS). The k-mesh is forced to be centered on the gamma point. The integration scheme for DOS uses Fermi smearing with a width of 0.05 eV. The absorption spectra were calculated using a time evolution algorithm [42].

## Data Availability

All data needed to evaluate the conclusions in the paper are present in the paper and/or the Supplementary Materials.
