# Supplementary material for: A High-Performance MoS2-Based Visible–Near-Infrared Photodetector from Gateless Photogating Effect Induced by Nickel Nanoparticles
Source: Research (Wash D C). 2023 Jul 14;6:0195. doi: 10.34133/research.0195 (PMC10348407; doi:10.34133/research.0195)
Supplement: Supplementary 1 — Figs. S1 to S9 [file research.0195.f1.docx]

**Supplementary Information for**

**A High Performance MoS_2_-Based Visible-Near Infrared Photodetector from Gateless Photogating Effect induced by** **Nickel Nanoparticles**

Ran Duan ^1^, Weihong Qi ^1,2*^, Panke Li ^1^, Kewei Tang ^1^, Guoliang Ru ^1^, Weimin Liu^1,3 *^

^1^ State Key Laboratory of Solidification Processing, Center of Advanced Lubrication and Seal Materials, Northwestern Polytechnical University, Xi’an 710072, China

^2^ Shandong Laboratory of Yantai Advanced Materials and Green Manufacturing, Yantai 265503, China

^3^ State Key Laboratory of Solid Lubrication, Lanzhou Institute of Chemical Physics, Chinese Academy of Sciences, Lanzhou 730000, China

^*^Correspondence should be addressed to Weihong Qi; qiwh216@nwpu.edu.cn and Weimin Liu; wmliu@licp.cas.cn


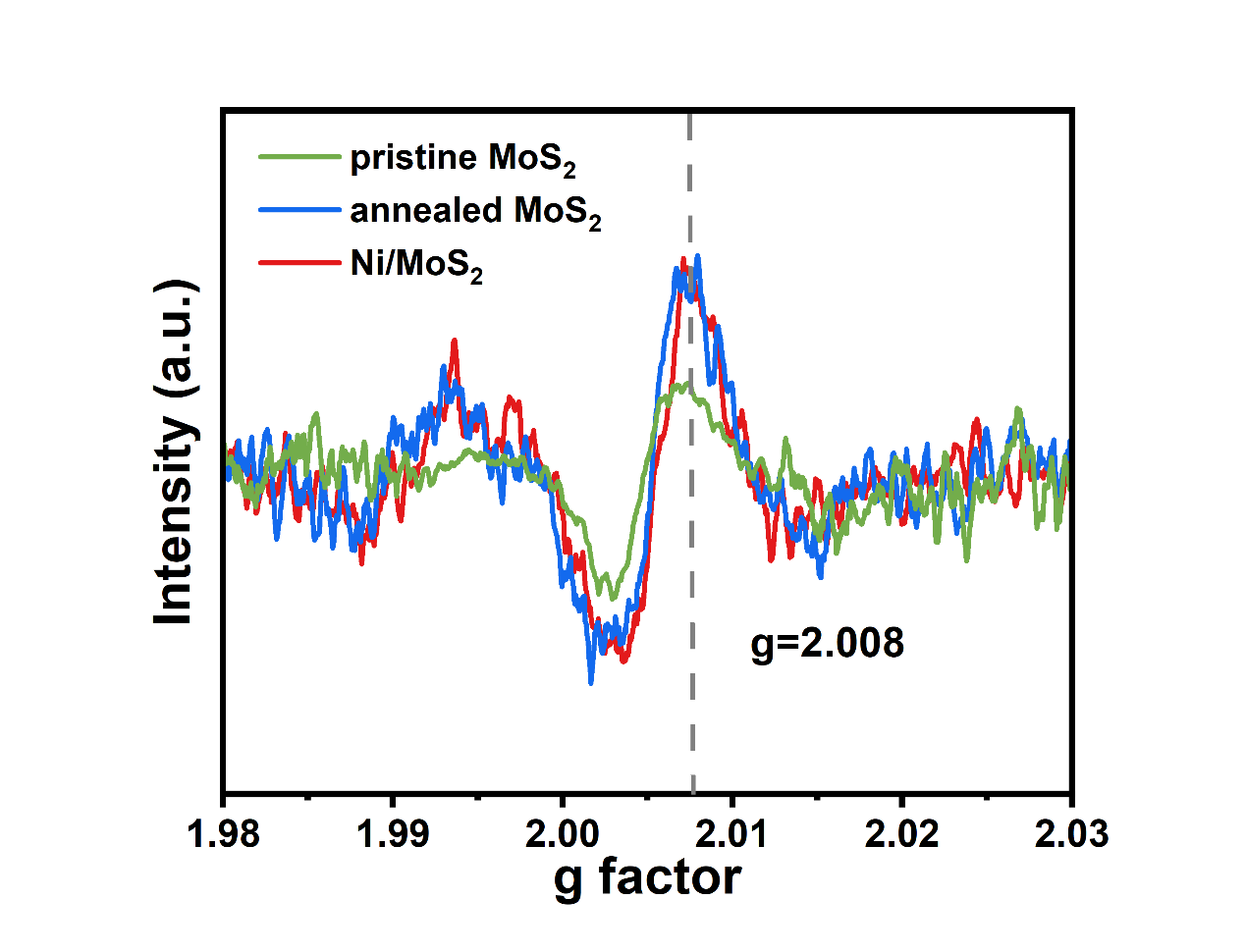


**Figure S1** EPR spectra of pristine MoS_2_, annealed MoS_2_ and Ni/MoS_2_.


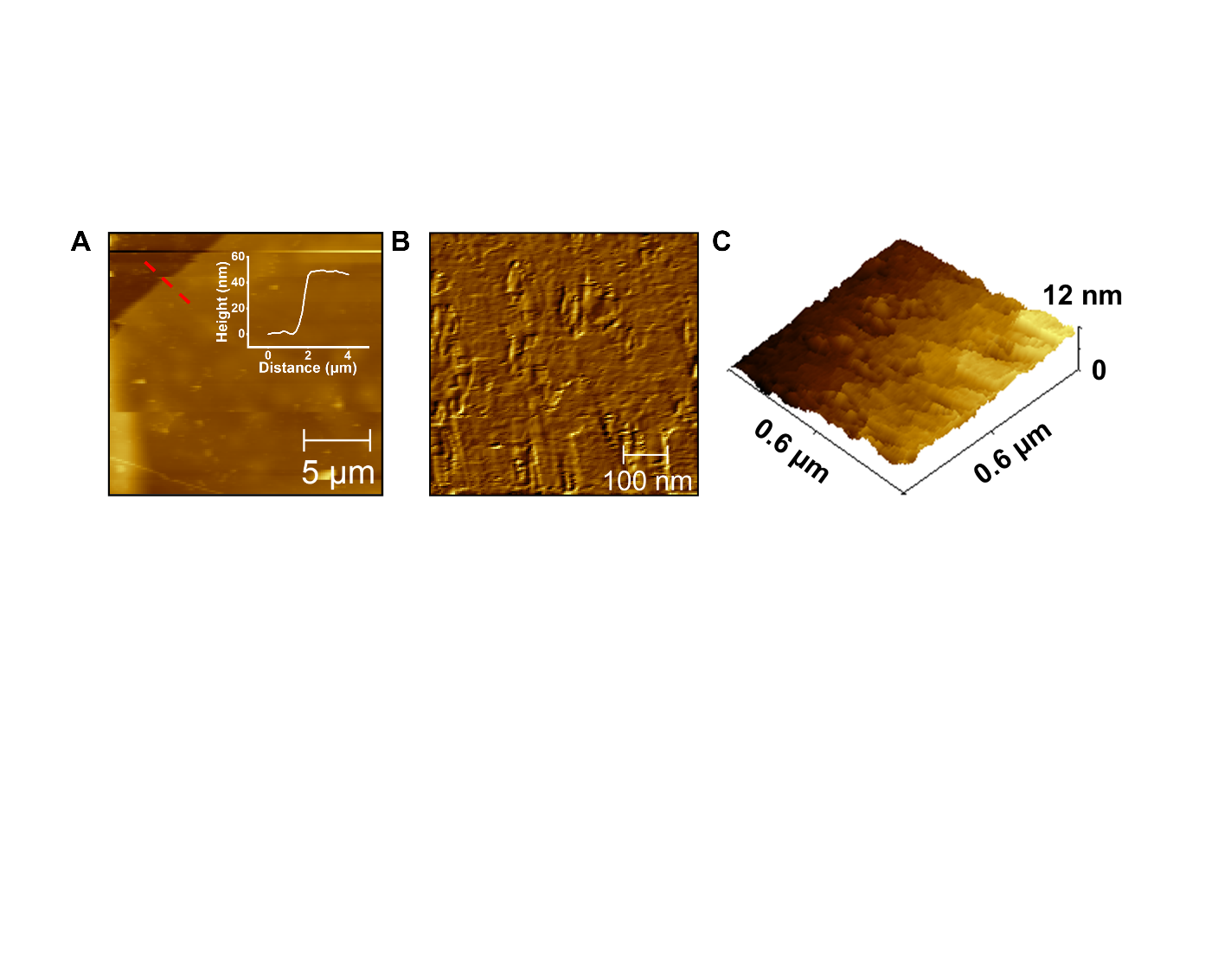


**Figure S2** (**A, B**) AFM image of MoS_2_ and (**C**) corresponding 3D view.


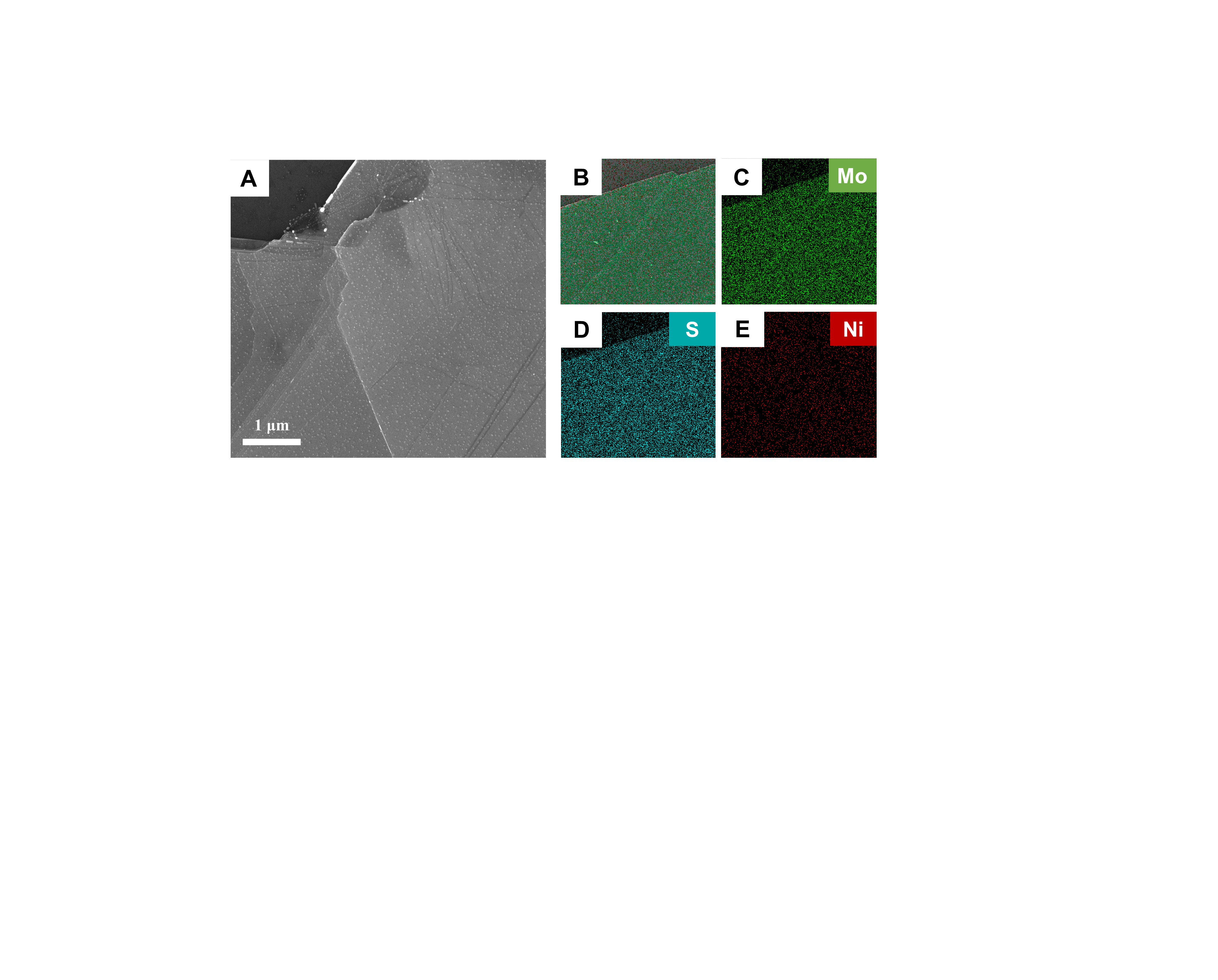


**Figure S3** (**A**) SEM image of Ni/MoS_2_; (**B**) EDS scanning area and elemental mapping of (**C**) Mo, (**D**) S and (**E**) Ni.


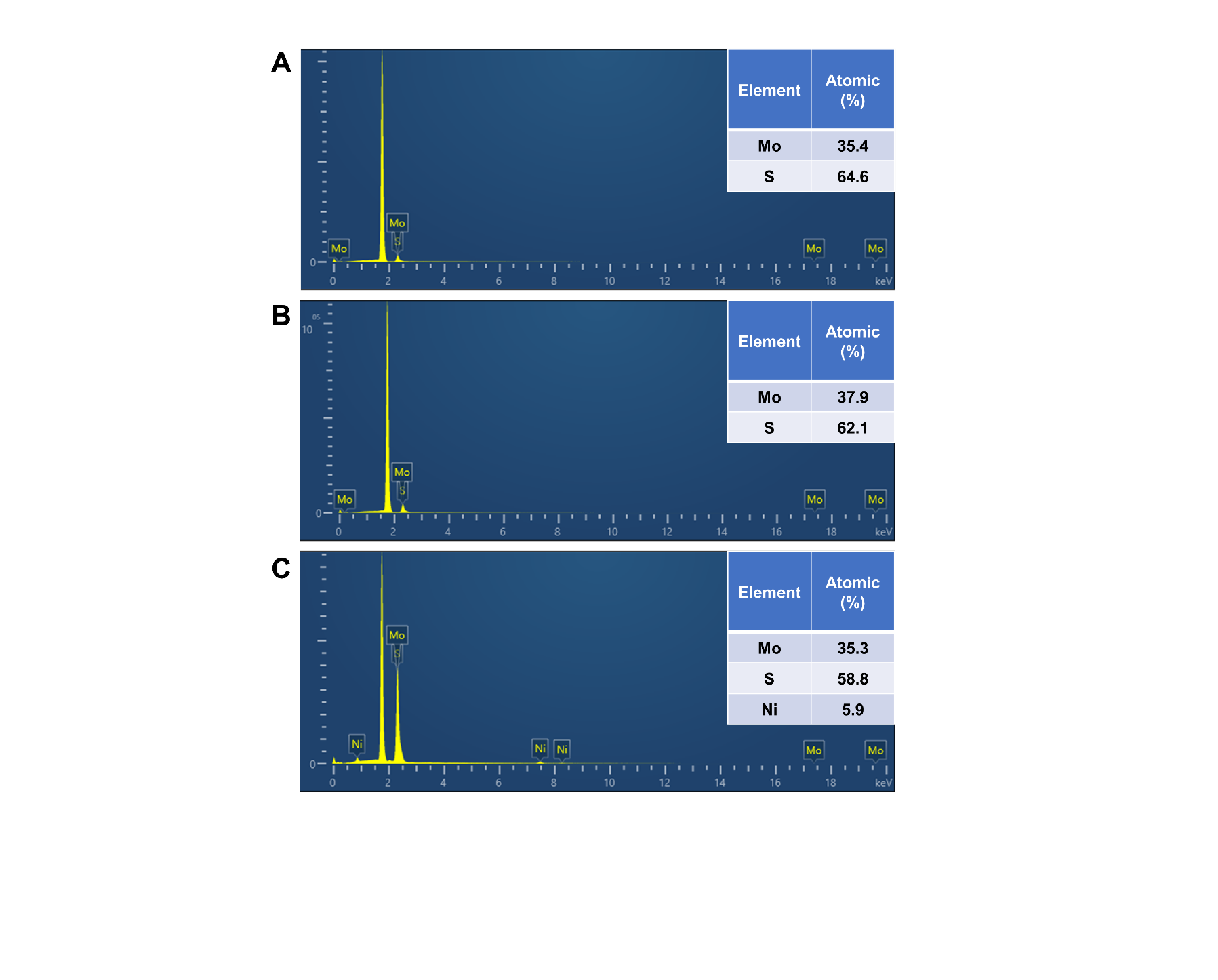


**Figure S4** EDS spectrum of (A) pristine MoS_2_, (B) MoS_2_ annealed in hydrogen atmosphere, (C)Ni/MoS_2_.


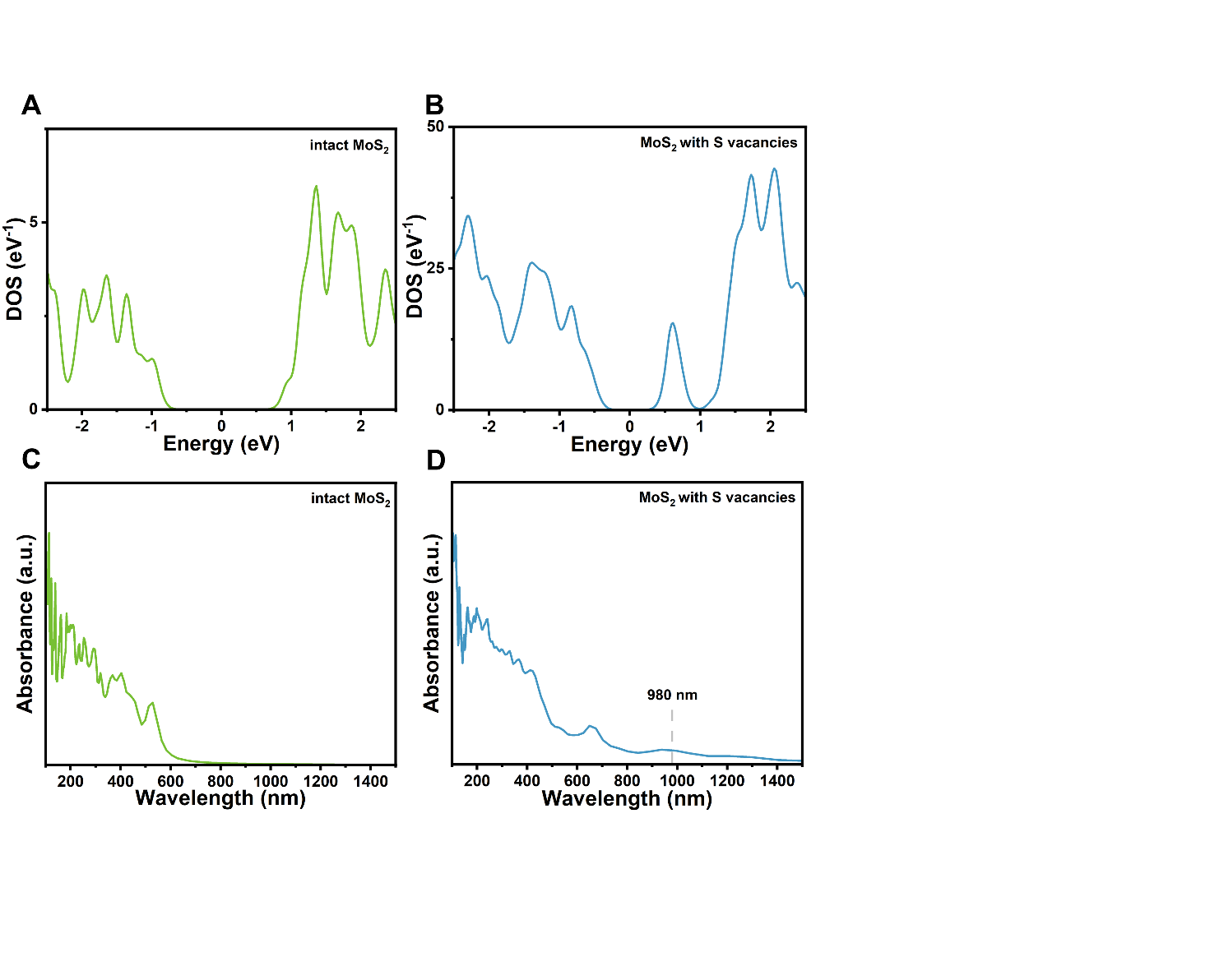


**Figure S5** Total density of states of (**A**) intact MoS_2_ and (**B**) MoS_2_ with S vacancies; the absorption spectra of (**C**) intact MoS_2_ and (**D**) MoS_2_ with S vacancies.

**
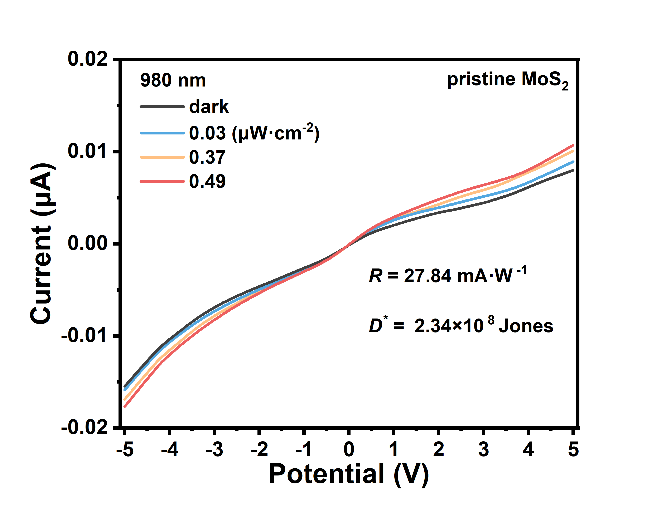
**

**Figure S6** The *I-V* curves and corresponding responsivity and detectivity of mechanically exfoliated MoS_2_ before annealing.


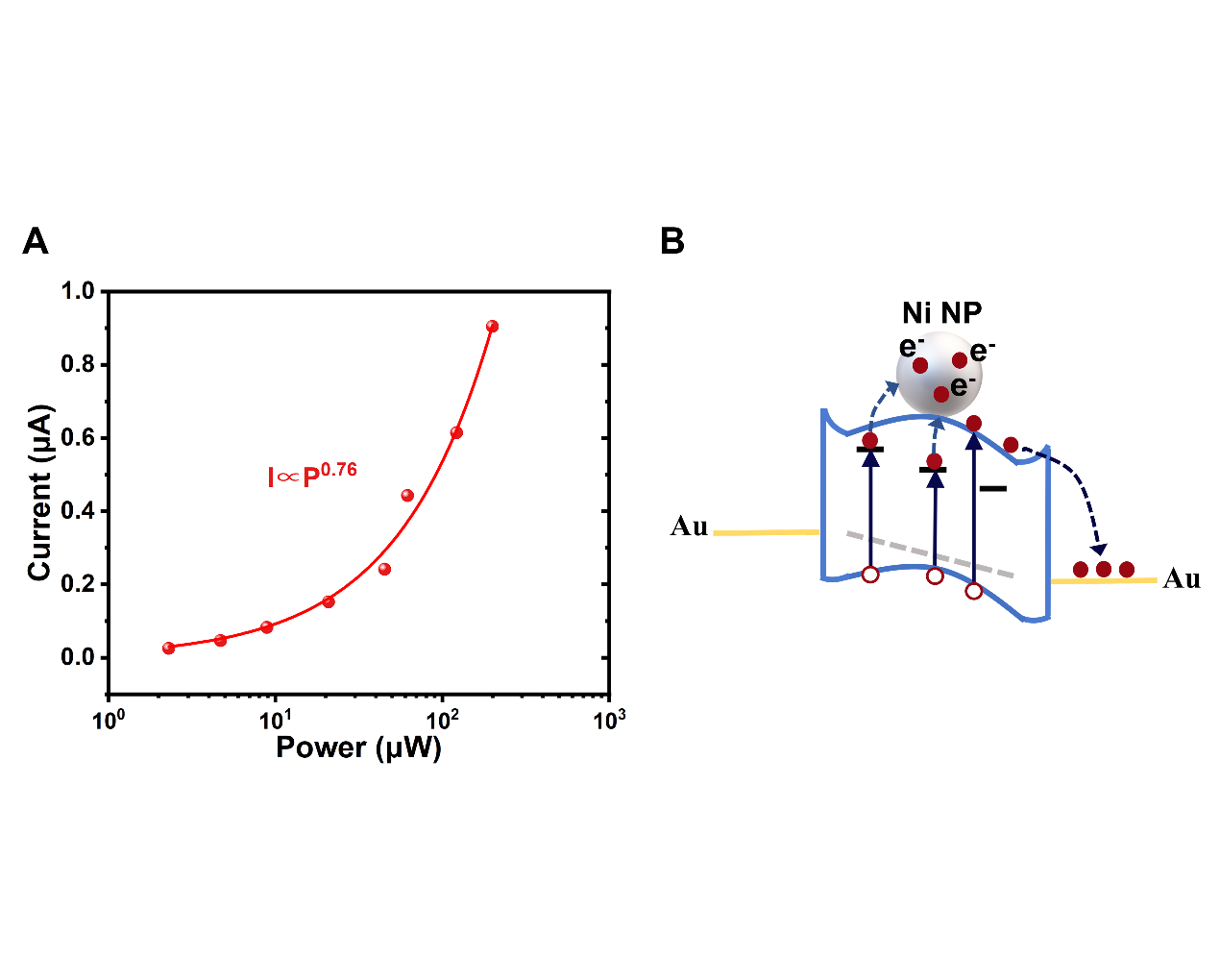


**Figure S7** (**A**) Photocurrent as a function of the incident light density of Ni/MoS_2_ at -5 V; (**B**) energy band diagram of Ni/MoS_2_ at negative voltage.


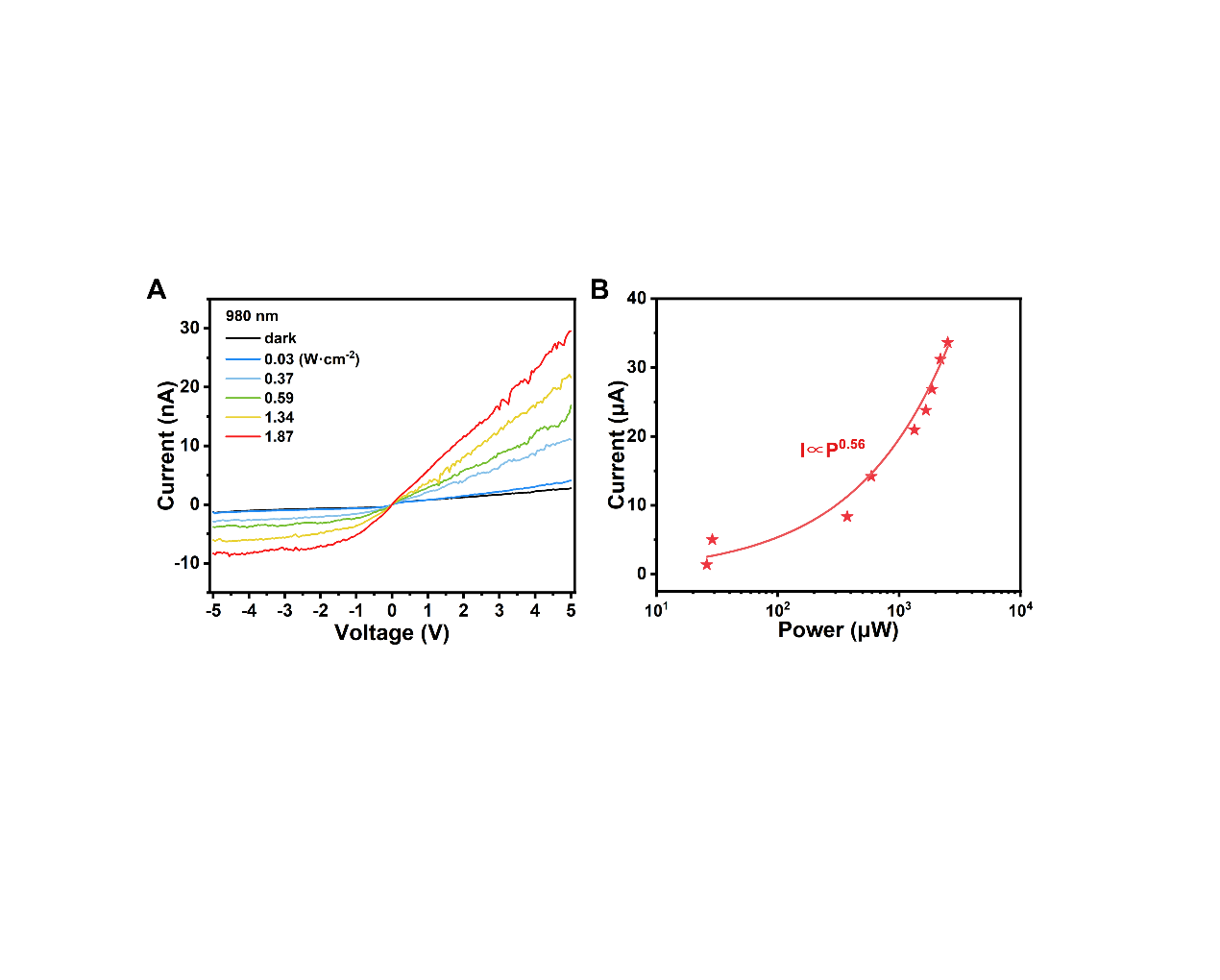


**Figure S8** (a, b) The I-V curves of the Ni/MoS_2_ device after eliminating Ni NPs by acid treatment under dark conditions and under 980 nm illumination.


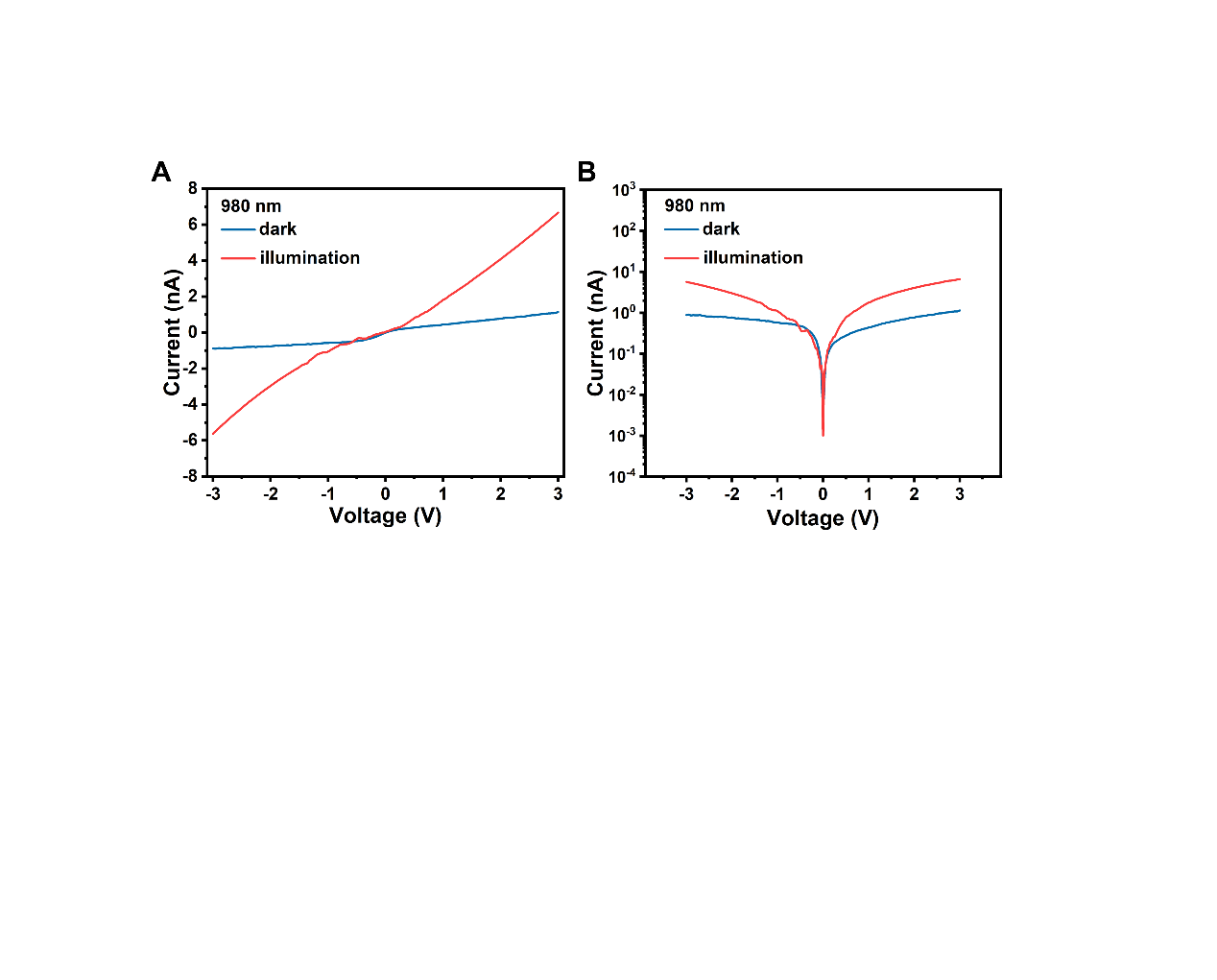


**Figure S9** (**A, B**) The I-V curves of the Ni/MoS_2_ device after eliminating Ni NPs by acid treatment under dark conditions and under 980 nm illumination.
